# Review: Myelin clearance is critical for regeneration after peripheral nerve injury

**DOI:** 10.3389/fneur.2022.908148

**Published:** 2022-12-16

**Authors:** YiMing Yuan, Yan Wang, ShanHong Wu, Ming Yue Zhao

**Affiliations:** ^1^Laboratory of Brain Function and Neurorehabilitation, Heilongjiang University of Chinese Medicine, Harbin, China; ^2^Department of Rehabilitation, The Second Affiliated Hospital of Heilongjiang University of Chinese Medicine, Harbin, China

**Keywords:** Schwann cell, myelin clearance, peripheral nerve injury, demyelination, autophagy, macrophages

## Abstract

Traumatic peripheral nerve injury occurs frequently and is a major clinical and public health problem that can lead to functional impairment and permanent disability. Despite the availability of modern diagnostic procedures and advanced microsurgical techniques, active recovery after peripheral nerve repair is often unsatisfactory. Peripheral nerve regeneration involves several critical events, including the recreation of the microenvironment and remyelination. Results from previous studies suggest that the peripheral nervous system (PNS) has a greater capacity for repair than the central nervous system. Thus, it will be important to understand myelin and myelination specifically in the PNS. This review provides an update on myelin biology and myelination in the PNS and discusses the mechanisms that promote myelin clearance after injury. The roles of Schwann cells and macrophages are considered at length, together with the possibility of exogenous intervention.

## Introduction

Injury to peripheral nerves leads to a series of molecular, cellular, and microstructural responses that promote their regeneration and functional recovery. Peripheral nerve regeneration may be hindered by slow organizational growth, degeneration of its distal segments, and impediments to its reinnervation. Regeneration is frequently insufficient and accompanied by negative clinical sequelae (1). While there has been substantial focus on clinical interventions designed to improve this process, the contributions associated with myelin clearance remain comparatively unexplored.

Lipid-rich myelin formed by Schwann cells (SCs) facilitates saltatory impulse transmission and provides trophic support to the peripheral nervous system (PNS) *via* radial sorting of its axons. SCs associate with and wrap around larger axons late in embryonic development to facilitate myelination that will be initiated postnatally. Regeneration of myelin is a critical aspect of any effective treatment for peripheral nerve injury (PNI). Remyelination is critical for effective repair after PNI and will be needed to provide new support for the PNS. Furthermore, myelin breakdown has been recognized as a key contributor to various peripheral nerve diseases.

Fragments generated by myelin breakdown obstruct axon regeneration. Myelin ovoids resulting from the destruction of the myelin sheath can be detected in SCs associated with degenerating axons (22). These ovoids contain numerous factors, including myelin-related glycoprotein (MAG) which can inhibit axon development (23, 24). Myelin debris at the lesion site results in local pressure and also impedes nerve repair (25). Jessen et al. reported that the PNS was able to undergo repair more effectively than the central nervous system (CNS) (26). This may be explained at least in part by its superior capacity for myelin removal (27). The results of several recent studies suggested that the removal of myelin debris is a prerequisite for effective nerve regeneration (28–30). Thus, an understanding of the mechanisms contributing to myelin removal from injured nerves will have broad implications for this process.

Earlier work has established that myelin breakdown occurs during the early stages immediately following nerve injury and is followed by the clearance of myelin debris. In this review, we will begin with a discussion of myelin formation. We will then discuss previous research and consider the results of recent studies that address the mechanisms involved in myelin removal. We will conclude with a discussion of the therapeutic implication of these findings.

The accumulation of myelin debris has a significant impact on nerve regeneration; this material is no longer useful and contains many inhibitory signals that prevent repair. Interestingly, and in contrast to the PNS, the CNS does not have satisfied mechanisms that promote effective myelin degradation (31). Myelin fragments can persist at the site of a CNS injury for months or even years where they serve to inhibit essential repair mechanisms (32). For example, Fujita et al. reported that MAG detected in CNS inhibits regeneration *via* its capacity to suppress tropomyosin receptor kinase (Trk) activity (33). In the nerve system, there is undoubtedly a balance between the clearance of myelin and detrimental consequences that impede regeneration, and this relationship seems to be better regulated in PNS.

**Figure 1:**
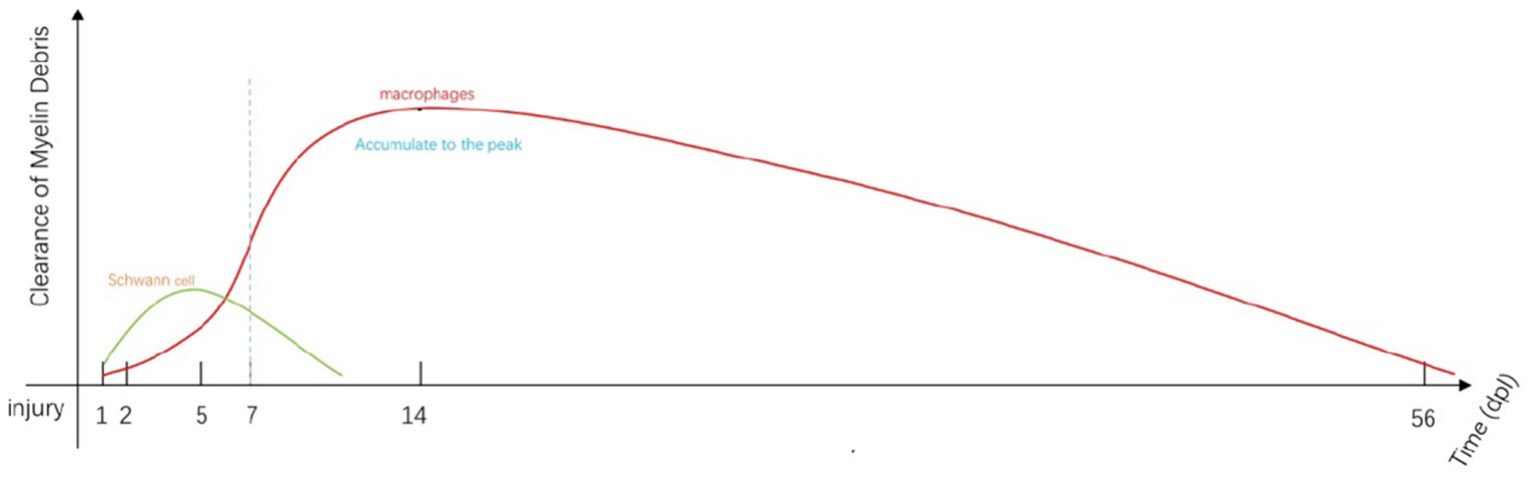
Immediately after injury, SCs and macrophages (including resident and early-stage infiltrating macrophages) begin to remove myelin debris (34). SC-mediated phagocytosis reaches a peak at approximately 5 days post-injury (dpi; green line). Most of the engulfment results from macrophage activity that increases from 5 to 7 dpi (red line). Macrophage-mediated phagocytosis reaches a peak at approximately 14 dpi and remains detectable at day 56 (35). Fibroblasts and neutrophils also contribute to myelin clearance at this stage (35, 36). The myelin fragments are ultimately cleared by localized apoptosis followed by transport by the lymphatics to the spleen.

In this review, we focus on new insights directed at the earliest responses to nerve injury (Figure 1); established mechanisms and conventional insights into this process have been reviewed elsewhere (37). The article highlights new findings that address various mechanisms that drive myelin removal and concentrates on new perspectives of peripheral nerve regeneration based on the construction of a suitable microenvironment. We also discuss the potential impact of myelin removal in other neurological diseases.

## The formation and function of myelin

Before discussing clearance, we need to have a full understanding of the mechanisms underlying myelin formation and function in PNS. Myelin is a lipid–protein complex derived from membranes of SCs (in the PNS) and oligodendrocytes (in the CNS) that forms a sheath that supports and protects individual axons (38), facilitates rapid transit of electrical signals, and provides critical nourishment. Myelin-providing (pro-myelin) SCs are generated from precursors that undergo three developmental shifts, including transitions from crest cells to SC precursors (SCPs), from SCPs to immature SCs, and from immature SCs to either pro-myelin SCs or non-myelinating (Remak) SCs. The pro-myelin SCs then undergo radial sorting; some of these cells go on to generate myelin sheaths (39, 40).

Myelin in the PNS is comprised of both lipids (70–80%) and proteins (20–30%) (41), the latter group including primarily myelin protein zero (MPZ), as well as myelin basic protein (MBP) and peripheral myelin proteins 2 and 22 (PMP2/PMP22). Those proteins undergo upregulation at the start of myelination and provide the myelin sheath with a unique membrane structure. The interactions between these proteins and the phospholipid bilayer result in compact cytoplasmic leaflets. Upregulated expression of these myelin genes is regulated by the POU-associated transcription factor, Oct6 (SCIP), Brn2, the high-mobility group protein Sox10, and the protein Krox20/Egr2 (42).

Both extrinsic and intracellular signals regulate the process of myelination. Among these signals, neuregulin (Nrg)1 regulates nearly all aspects and functions of SCs (40). Similarly, laminin and laminin receptors modulate myelination *via* their role in promoting essential autocrine signals (43). Likewise, Gpr126 provides critical signals that promote myelination *via* its role in regulating signaling by cyclic adenosine monophosphate (cAMP) (44, 45). Myelination also relies on several downstream intracellular signaling pathways involving PI3K, PLC-γ, focal adhesion kinase (FAK), mitogen-activated protein kinase (MAPK), and Wnt/β-Catenin (46–48). In addition, cAMP promotes and maintains myelination *via* both intracellular signaling as well as mechanisms that regulate its expression (49, 50).

Results from previous reports revealed that iron was required for myelin production and its maintenance in the CNS (51, 52). Results from more recent studies revealed that iron homeostasis is also required for myelin formation in the PNS; Santiago González et al. reported the divalent metal transporter 1 (DMT1) (53), heavy ferritin chain (Fth), and the transferrin receptor 1 (Tfr1), which are factors that regulate iron absorption and storage, provide crucial contributions to SC maturation and myelin formation. Iron outflow also has an impact on myelin formation. The ferroxidase enzyme, ceruloplasmin (Cp), protects the PNS and regulates iron efflux, thereby modulating the maturation of SCs and axon myelination (54). Disorders of iron homeostasis can induce oxidative stress and SC injury (53, 54).

A comprehensive understanding of the composition of myelin and the mechanisms involved in its formation will be essential for our assessment of myelin clearance that takes place in response to nervous system injuries. We note that the factors and signals described earlier also play critical roles in regulating myelin removal after injury. Furthermore, the connections linking myelin formation and removal have important implications with respect to the treatment of PNI.

## Negative regulation of myelination

**Figure 2:**
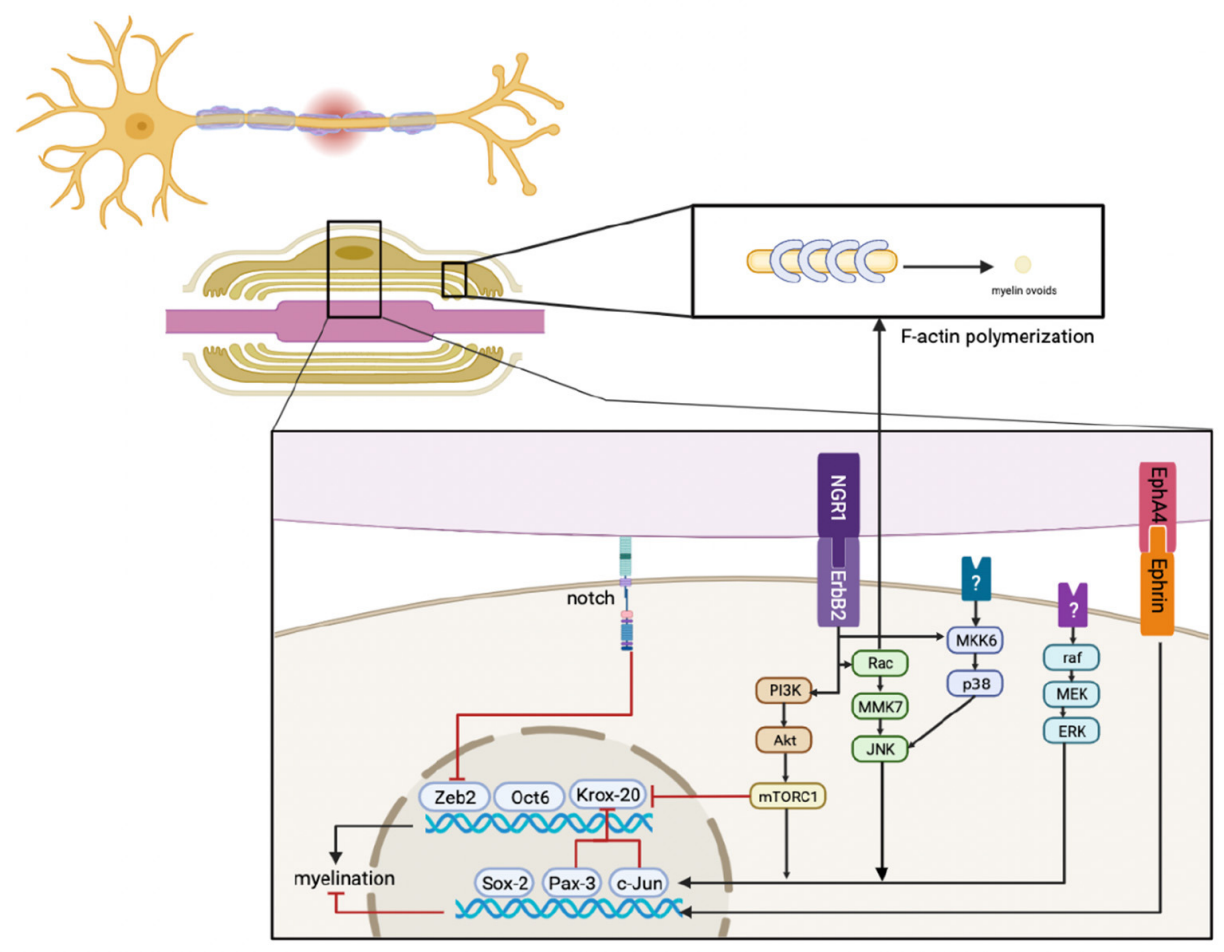
Immediately after injury, ErbB2, MAPK, PI3K, Notch, and EphA4 signals respond in SCs, their crosstalk, and the downstream signaling pathways that regulate factors that mediate myelination. This results in the downregulation of pro-myelinating genes, including Zeb2, Oct6, and Krox20, and the upregulation of negative regulatory factors, including Sox-2, Pax-3, and c-Jun. In addition, activated Rac promotes F-actin polymerization to generate myelin ovoids. Myelin is also cleared during this stage.

Demyelination is a characteristic response to a peripheral nerve injury. Pro-myelin SCs activate a cellular program that leads to the disintegration of the myelin sheath. Demyelination of injured peripheral nerves typically takes place *via* Wallerian degeneration (WD) that both negatively regulates myelination and leads to myelin degradation. Reduced expression of myelin-associated proteins may also contribute to rapid demyelination, as this response promotes SC proliferation, clearance of myelin debris, and nerve regeneration (9). We will thus review this process as the first step of the clearance program, as this approach can help us to understand the molecular mechanisms that promote the clearance of myelin sheath fragments (Figure 2).

### Factors that regulate myelination

#### c-Jun

The transcription factor, c-Jun, is found at the core of the AP-1 complex and is involved in the formation of the myelin sheath (55). More recent evidence has elucidated the role of c-Jun-mediated activities that regulate the responses of denervated SCs after injury, notably the formation of regeneration tracks, support of neuronal survival, promotion of axon regrowth, and myelin clearance (56). Furthermore, c-Jun deletion resulted in diminished clearance of myelin fragments and delayed regeneration (56). Additional evidence revealed that diminished quantities of O-GlcNAc transferase (OGT), which is an enzyme associated with post-translational modifications that suppress c-Jun, also impaired the process of remyelination (57); this result also suggested that activated c-Jun was an essential component of the regeneration process. Results from additional studies revealed that AMP-activated protein kinase (AMPK) attenuated myelin gene expression and thus reduced myelin formation, which is also a response to the activation of c-Jun signaling (58). SC dedifferentiation, myelin clearance, and regeneration were all impaired in the absence of c-Jun (56, 59). While overexpression of c-Jun can also harm the repair, process, and moderate activation is required (60).

#### Sox2

The Sox2 transcription factor regulates the activities of Schwann cells. Earlier *in vitro* studies revealed that Sox2 negatively regulated both crest and stem cells and thus regulated the formation of myelin (61). Recent findings have revealed that Sox2 may have a specific impact on myelin produced in the PNS. Interestingly, sustained expression of negative regulators, including Sox2 and Id2, protected mice modeling Charcot–Marie–Tooth (CMT) 1B disease from both dysmyelination and the deleterious impact of mutated myelin-related proteins, including MPZ (62). Activation of SC-autonomous and SC-nonautonomous protective functions may be a key mechanism underlying this result.

#### Pax-3

Pax-3 is a paired domain transcription factor expressed in the PNS and SCs that represses the induction of MBP by preventing the activation of the MBP promoter by cAMP (63, 64). Furthermore, Doddrell et al. (65) reported that Pax-3 strongly inhibited the cAMP-mediated induction of both Krox20 and Oct-6 in SCs as well as the expression of Sox-10. Interestingly, Pax-3 inhibited the expression of c-Jun and enhanced Notch-1 expression (65); collectively, these results suggested that Pax-3 inhibited Krox20 in a c-Jun-independent manner. However, while these factors are all involved in the repression of myelination, the precise mechanisms will need to be elucidated by further research.

### Pathways regulating myelin gene expression after injury

#### MAPK and NRG1

ERK signaling is strongly and rapidly activated approximately 4 h after insult at both injured and distal sites (66, 67). Harrisingh et al. (67) described the role of the Raf-ERK pathway in promoting SC dedifferentiation. As a first step, Raf activation suppresses the expression of myelin-associated proteins induced by cAMP (67, 68). Moreover, results from another previous study revealed that Raf overexpression generated in response to tamoxifen strongly activates Raf-ERK signaling and downregulates myelin-related gene expression in an uninjured nerve (69). Sustained activation of ERK also resulted in morphological defects, restricted regeneration, and impaired functional recovery, although rapid demyelination was observed (70).

Crosstalk between NRG1 and MAPK signaling pathways promotes demyelination and is a crucial factor involved in the migration of crest cells and the generation of mature SCs, as well as maintaining appropriate myelin thickness (71–73). Although NRG1 promotes both proliferation and the formation of myelin (50), recent results suggest that NRG1 may also promote reductions in the concentration of myelin fragments (73). The role of Rac-MAPK in the processes underlying demyelination is somewhat better clarified and is regulated by Ngr1/ErbB2 signaling (74, 75). Its intracellular target, MAPKK7 (MKK7), is upstream of JNK and was reported to induce the expression of c-Jun as opposed to ERK (76). The net result was the downregulation of myelin gene expression at the distal site of an injured nerve.

Yang et al. reported that p38 MAPK was responsible for NRG1-mediated downregulation of myelin as well as the blockade of Krox20 expression. Results from this report also revealed that activated MKK6, which is a direct and specific upstream activator of p38 MAPK, also promoted demyelination (77).

Several additional factors related to this pathway were also found to delay myelination. Among these, bone morphogenetic protein (BMP)7 limited the formation of myelin sheath during development (78). Dummula et al. reported that the inhibition of BMP expression resulted in myelin preservation in the CNS; these events also had an immediate impact on the maturation of oligodendrocyte precursor cells and myelin formation (78). Recent evidence suggests that BMP family proteins exhibit a similar function in PNS. Results in this report revealed that BMP4 was upregulated beginning at 12 h after injury and reached peak expression at 24 h; expression then decreases continuously thereafter (79), paralleling demyelination. Further analysis revealed that BMP resulted in reductions in cAMP-induced expression of myelin genes, potentially due to its capacity to target the p38MAPK/c-Jun axis. Similarly, fibroblast growth factor 21 (FGF21) was originally identified as a factor that promoted remyelination in CNS (80). However, recent results suggest that FGF21 has completely different functions in PNS, specifically those resulting in the repression of myelin-related genes (81). The results from this report revealed that negative regulation secondary to FGF21 was mediated by the p38 mitogen-activated protein kinase (MAPK)/c-Jun axis. Zhang et al. reported that the expression of FGF21 in developing SCs was diminished in the presence of dibutyryl-cAMP, thus suggesting dynamic regulation of myelin production (81). SCs in the PNS can synthesize and secrete FGF21 which extends its impact beyond the paracrine observed in the liver. Therefore, FGF21 and BMP7 may be among the critical negative regulatory factors involved in myelination after nerve injury. Interestingly, Wang et al. reported the increased expression of genes associated with immature SCs and decreased expression of genes associated with mature SCs in FGF9-gene-deleted mice (82). Overall, these results suggest that FGF9 may also seem to be involved in the process of myelin degeneration.

The intracellular GTPase Rac regulates actin polymerization and is a component of an essential pathway involved in the fragmentation of myelin (74, 83). Myelin ovoids formed during the early stages of WD may be responsible for the SC-mediated removal of compact myelin sheath material (84). After an injury, WD begins with the stereotypic fragmentation of myelin sheath into myelin ovoids at the Schmidt–Lanterman cleft (SLC), which is an area that contains non-compact myelin sheath material. Then, the structure of the SC cytoskeleton changes considerably. Although the SLC features atypical adherens junctions (AJs) (48, 85), the molecular nature of SLC appears to parallel those, and polymerization of new actin filaments occurs after injury and promotes the degeneration of both AJs and E-cadherin, thereby inducing the myelin fragmentation (74). Activated Rac GTPase localizes to the SLC and regulates actin polymerization. By contrast, inhibition of actin polymerization also suppressed the formation of myelin ovoids and the degeneration of E-cadherin. Thus, we conclude that new F-actin that polymerizes after WD promotes the clearance of myelin sheath material (83).

#### Notch signaling

Notch is a transmembrane receptor protein that is cleaved to generate a Notch intracellular domain (NICD) after combining it with its ligand, Jagged-1. Notch signaling is a central pathway in developing invertebrates that are upregulated in response to Sox-2 and downregulated in response to Krox20 signaling. NICD preserves myelination and induces its expression (50).

Results from previous research revealed that activated Notch pathways regulated demyelination and proliferation of SCs (86); engineered overexpression of NCID results in rapid demyelination of injured nerves (87). Wang et al. reported that forced overexpression of NCID promotes peripheral nerve regeneration *via* pathways that promote accelerated demyelination (82). Moreover, Notch acts as the inhibitor of Zeb2 (88) and is thus involved in another essential pathway associated with myelin regeneration (89).

A recent study reported that post-translational sumoylation negatively regulates angiogenesis *via* its impact on Notch signaling (90). Accordingly, one might speculate that sumoylation might also have a negative impact on myelination *via* its capacity to target the Notch signaling pathway in SCs (91). Sumoylation may also inhibit Oct6 and Krox20 (11), although the impact of these modifications on myelination remains unexplored.

#### PI3K-Akt-mTORC1

mTORC1 is a central modulator of numerous anabolic reactions and promotes myelination in the PNS (60). Interestingly, mTORC1 undergoes transient activation after injury and suppresses myelination (92). mTORC1 is activated in SCs after injury *via* NRG1-PI3K-Akt signaling that suppresses myelination rather than promoting regeneration (53, 93, 94). Activated mTORC1 has no impact on macrophage recruitment (60). mTORC1 also promotes demyelination by inhibiting Krox20 and activating c-Jun (60, 93). However, mTORC1-mediated demyelination was not enhanced *via* the additional activation; this may be due to feedback inhibition on the PI3K-Akt pathway (94). Over-activation of mTORC1 also resulted in delayed regeneration; this dual function may be a new target for therapeutics designed to combat nervous system diseases.

#### Neuregulin1

Neuregulin1/ErbB2 signaling pathways are involved in crosstalk with MAPK and PI3K and regulate demyelination. NRG1 is crucial to the formation of glia and the migration of crest cells as well as the formation of mature SCs and mechanisms that control myelin thickness (71–73). The results of several previous studies revealed that NRG1/ErbB signaling could target the bZIP transcription factor Maf, thereby limiting the formation of myelin *via* its impact on cholesterol levels (57, 95). Therefore, NRG1 signaling is strongly connected to myelin clearance.

Recent reports have revealed that the expression of soluble NRG1 changes in response to peripheral nerve injury and ultimately downregulates the expression of myelin-related genes including Pmp22, Serinc5, Ndrg1, Fa2h, Mal, Rxrg, and Krox20, in response to injury and activation of c-Jun (96). NRG1 responses during the first stage of clearance regulated myelin-associated gene expression. By contrast, NRG1 promotes myelin regeneration during the following stages (and also during development); these results suggest that NRG1 also acts as a dynamic regulator that stabilizes these processes with respect to the pathophysiological responses of the PNS. The interactions between these mechanisms permit myelin clearance to proceed.

The NRG1 type III (NRG1 III) isoform positively regulates the formation of myelin and radial sorting together with input from laminin α2β1γ1 (Lm211) (97, 98). Recently, laminin α2β1γ1 was identified as playing a negative role in the regulation of myelin formation in SCs (99). Likewise, tumor necrosis factor (TNF)-α-converting enzyme (TACE; also known as ADAM17) was found to cleave NRG1 III within its epidermal growth factor domain which then negatively regulates the formation of the myelin sheath (100).

#### EphA4

Eph/Ephrin is an important neuronal mediator that is responsible for glial activation, axon guidance and regeneration, and synapse plasticity and formation (101), as well as the regulation of myelin formation (102). EphA4 regulates CNS myelination *via* the ephrin-A1-EphA4 pathway (103). More recent research suggests that Eph4 negatively regulates the formation of myelin in SCs (104). The results of this study revealed upregulation of EphA4 until 14 days after injury; expression of both MAG and MPZ decreased after peripheral nerve injury, consistent with a role for EphA4 in inhibiting myelin sheath formation. The observed suppression of remyelination may be the result of SC proliferation during the early phases after injury and suppression of the differentiation of SCs at a later stage (40, 104, 105). Although this mechanism inhibits regeneration for an extended period (until 28 days) after injury, its potential to accelerate myelin clearance must also be considered. Collectively, these results suggest that appropriate negative regulation of myelination is beneficial to the regenerative process. Although the mechanisms remain to be clarified, factors that regulate the expression of EphA4 may emerge as new targets for drugs designed to promote myelin removal.

#### Transient receptor potential vanilloid 4 channels

Transient receptor potential vanilloid 4 is a non-specific calcium-permeable cation channel that is expressed widely and activated throughout the nervous system. One recent study revealed that TRPV4 promoted regeneration *via* demyelination of static nerves 2 to 14 days after injury (106). Interestingly, Remak SCs express high levels of TRPV4. Notably, TRPV4 gene-deleted mice exhibit delayed demyelination, thereby repressing nerve regeneration and delaying functional recovery. Moreover, the expression of TRPV4 undergoes a significant increase and returns to normal levels at 21 days due to the rapid myelination that takes place during the stage to follow (106). TRPV4-mediated alterations in calcium flux may have an impact on this process (107, 108). Therefore, TRPV4 and/or related calcium channels may be new targets used to treat PNI.

Because demyelination and regeneration are temporally correlated to one another, demyelination may be one factor contributing to the mechanism underlying nerve regeneration. WD and inhibition of myelin regeneration at early stages both have a positive impact on myelin clearance. Furthermore, some signals are dual-functioning with respect to myelin generation; these signals might be explored as a means to promote more effective regeneration. However, the basis of these interactions and/or synergy needs to be investigated further to promote effective treatments of neurological diseases.

## Autophagy and myelin clearance

Autophagy is an important pathway used to dispose of and ultimately recycle disordered proteins and discarded cellular structures and is critical for maintaining cell stability (109). At its most basic level, autophagy is necessary to preserve the integrity of cellular structures. The process involves five main steps, including (1) initiation and nucleation, (22) elongation of the membrane, (23) cargo sorting, (24) fusion of the mature autophagosome with lysosomes, and (25) nutrient recycling and renewal (110). Autophagy is one of the critical processes used by SCs to clear tissue debris that accumulates after PNI.

### Autophagy in SCs: A crucial process

The results of a recent study by Jang et al. (111) revealed that the primary myelin ovoid contains a fluid-filled axoplasm with a compact myelin sheath and scanty abaxonal cytoplasm that form P (proximal)-fibers. The P-fibers were eventually overtaken by D(degeneration)-fibers, which have been described as hypertrophic SCs that contain several small myelin vesicles associated with the process of degeneration. Agents that inhibit the function of lysosomes delay the transition from P- to D-fibers. These findings revealed that autophagy is essential for the timely clearance of the myelin sheath after PNI; interestingly, this process cannot be completely accomplished by macrophages recruited from circulation. Thus, autophagic SCs are needed to promote the earliest stages of myelin clearance, myelin regeneration, and scar reduction (112).

**Figure 3:**
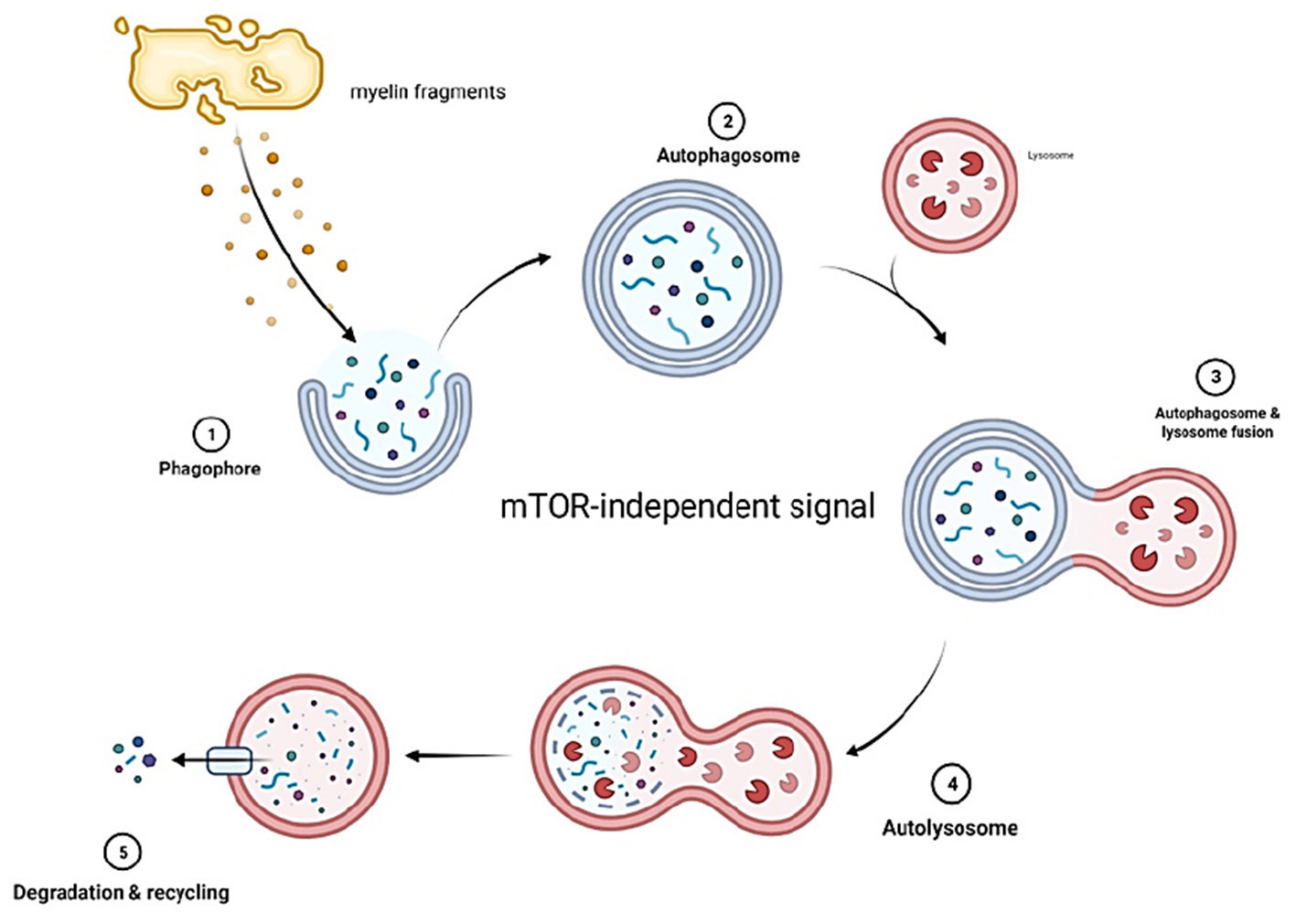
Myelin fragments at the site of injury form phagophores which develop into autophagosomes. The autophagosomes then fuse with lysosomes that contain enzymes capable of digesting myelin fragments. The decomposed fragments are then released into circulation. Autophagy in SCs is mTOR independent.

Autophagy in SCs is activated during development and suppressed once the cells reach a mature stage (111, 113). Although autophagy will be suppressed at maturity, SCs still utilize this function to eliminate unnecessary cytoplasm, thereby maintaining homeostasis (Figure 3).

SCs will rapidly switch their transcriptional program to a repair state that promotes the early stages of myelin clearance in response to PNI. Activation of autophagy is a critical component of this transformation (114). Autophagic clearance mechanisms improve the microenvironment, provide basal energy for SC survival (104), and prevent the emergence and recurrence of neuropathic pain (115). Notably, lipids represent 70–80% of the composition of peripheral myelin (116) and lipid droplets have been detected as autophagic cargo (117). Therefore, autophagy is a crucial process involved in nerve repair; impaired autophagy results in reductions in both myelin degradation and remyelination (118).

### Main regulators of autophagy and autophagic flux

#### Nerve growth factor

Li et al. reported that levels of autophagy-related (ATG)-7, ATG-5, Beclin-1, and LC3 proteins all increased in response to treatment with exogenous nerve growth factor (NGF) (119). In contrast to the traditional perspective, recent results suggest that the administration of NGF upregulates autophagic activity in SCs, most likely *via* the p75NTR/AMPK/mTOR pathway. In addition, NGF binds to p75NTR and activates small GTPases to increase the expression of c-Jun. Augmented expression of c-Jun might inhibit the myelination as described earlier. Therefore, the pathways linking NGF and autophagy specifically in SCs may represent novel therapeutic targets for drugs designed to promote nerve regeneration.

#### SIRT1/Hypoxia-inducible factor 1α

The NAD-dependent deacetylase, Sirt1, plays a critical role in regulating autophagy (120). Recent evidence suggests that overexpression of Sirt1 promotes regeneration and functional recovery *via* the activation of autophagy (121). Sirt1 promotes mTOR-independent autophagy *via* the activation of the downstream factor, Hif1α (121). Interestingly, these results suggest that Sirt1 might drive autophagy directly *via* its capacity to inhibit both mTOR and FOXO (122). Although the precise mechanism involved in this pathway requires further elucidation, it may also represent a target for one or more novel treatment strategies.

#### CXCL12

CXCL12 is one of the most widely studied chemokines and binds to its specific receptor, CXCR4/7. Administration of CXCL12 elicited positive therapeutic effects in various nervous system diseases (123–126). Of note, CXCL12 enhances autophagy after nerve injury and thus promotes nerve regeneration (126). Specifically, recombinant CXCL12 enhances autophagy in SCs in a time-dependent manner and inhibits the PI3K/AKT/mTOR pathway. Thus, the increased levels of autophagy induced by CXCL12 may be related to its capacity to inhibit mTOR (126).

#### Calcineurin

Calcineurin is a calcium-dependent serine/threonine protein phosphatase that is composed of calcineurin A (CnA) and calcineurin B (CnB). Cn was originally identified as a regulator of autophagy in lysosomal biogenesis (127). Recent findings reveal that Cn regulates autophagy *via* the activation of a calcium signaling pathway and transcriptional factor EB (TFEB) (127, 128). Additional evidence documented the role of exogenous trehalose in inducing autophagy in models of motor neuron degeneration (129); Reed et al. reported that CnB might play a critical role in this pathway. Furthermore, CnB gene-deleted mice exhibited a reduced autophagic influx, delayed myelination, and altered radial sorting (130). CnB ablation also impaired translocation to the nucleus, suggesting that Cn was capable of activating autophagy in SCs *via* the actions of TFEB.

Interestingly, Cn had no significant impact on the development of myelin (130); likewise, most of the other factors that regulate myelin clearance are also not closely associated with the process of myelin formation.

#### Vacuolar protein sorting family

PI3K vacuolar protein sorting 32 (Pik3c3) contributes to the synthesis of PI3P and is thus crucial for membrane transport and autophagy. The complex nature of PI3P regulation has been considered one of the mechanisms associated with CMT disease. In one recent study, Logan et al. found that mature autophagosomes could not be formed in Vps34-deficient SCs in the absence of PI3P, and thus the process of autophagy would be destroyed (131).

Wang et al. have recently suggested the existence of an ESCRT-Vps4-autophagy pathway that regulates the clearance of cellular debris (132). Vps4 is an ESCRT accessory protein that plays a vital role in regulating axonal autophagic flux. Cellular levels of Vps4 were found to influence the autophagic flux in experiments performed in *Drosophila melanogaster*. Subsequent studies performed in mammalian models revealed that Vsp4 provided support to the autophagic flux but was not critical for its induction (132). Vps4 was consumed rapidly after injury and may induce the accumulation of autophagosomes and thus impair cellular organization. Therefore, we speculate that Vps4 may have the capacity to promote nerve regeneration *via* the regulation of autophagy and thus the clearance of myelin debris.

#### FGF

The basic fibroblast growth factor (bFGF) is a major neurotrophic factor secreted by SCs as well as neuronal cell populations. High levels of bFGF expression can promote numerous functions associated with PNI, including neuroprotection, neurogenesis, and the generation of an appropriate microenvironment (133, 134). Notably, recent studies revealed that the upregulation of bFGF promotes myelin clearance *in vitro* via the maintenance of an effective autophagic flux at the early stages after PNI (135). Additional studies suggested that bFGF most likely targets the TFEB pathway (135).

In addition, recent evidence suggests that fibroblast growth factor 1 (FGF1) elicits its protective and regenerative effects on SCs due to its capacity to regulate autophagy *via* peroxiredoxin (PRDX1), possibly in association with the Wnt pathway (136).

Unlike the classical activation of autophagy, which relies on the inhibition of the mTOR pathway, autophagy in SCs is activated by an mTOR-independent mechanism that is regulated by the actions of ceramides on the JNK/c-Jun pathway (114, 118, 137). Activation of this pathway may be synergistic with the inhibitory effect of c-Jun on myelin-associated protein expression in response to acute injury. Interestingly, mTORC1 is necessary for myelin clearance during the post-injury period *via* its role in promoting the dedifferentiation of SCs and increasing the expression of c-Jun, thereby inhibiting the expression of myelin-related proteins (60). Collectively, these findings may explain how SC-mediated autophagy occurs *via* an mTOR-independent mechanism.

## Other SC-associated mechanisms that promote myelin clearance

### TAM receptor tyrosine kinases

Tyro3, AXL, and Mer are among the TAM receptors. Members of this family of receptor tyrosine kinases promote SC-mediated phagocytosis *via* mechanisms that are similar to those used by macrophages. Interestingly, mouse model strains with defective autophagy remain capable of debris clearance over an extended period. These observations imply the existence of other pathways that can compensate for one or more autophagic defects. Recent evidence from mouse models of PNI suggests that SCs may promote clearance *via* TAM receptor (Axl/Mertk)-mediated phagocytosis (138). Clearance of myelin debris was impaired in mice devoid of these receptors.

#### Transcription factor nuclear factor erythroid-2-related factor 2

Transcription factor Nrf2 is activated in response to oxidative stress that develops after PNI. Oxidative stress typically results from a crush injury or chronic neuronal constriction; reactive oxygen species (ROS) are also produced after a transection injury (139–141). Nrf2 is held in the cytoplasm by its Kelch-like ECH-associated protein 1 (Keap1) inhibitor domain. Gene expression is activated *via* its antioxidant response elements (AREs) (142). When compared to wild-type mice, Nrf2-deficient strains were present with more myelin residue, lower levels of macrophage infiltration, and weak recovery of the neuromuscular junction. Thus, there may be a critical role for a Keap1-Nrf2-ARE pathway (143), and Nrf2 may be a crucial factor involved in promoting the timely clearance of myelin fragments.

## Macrophages

**Figure 4:**
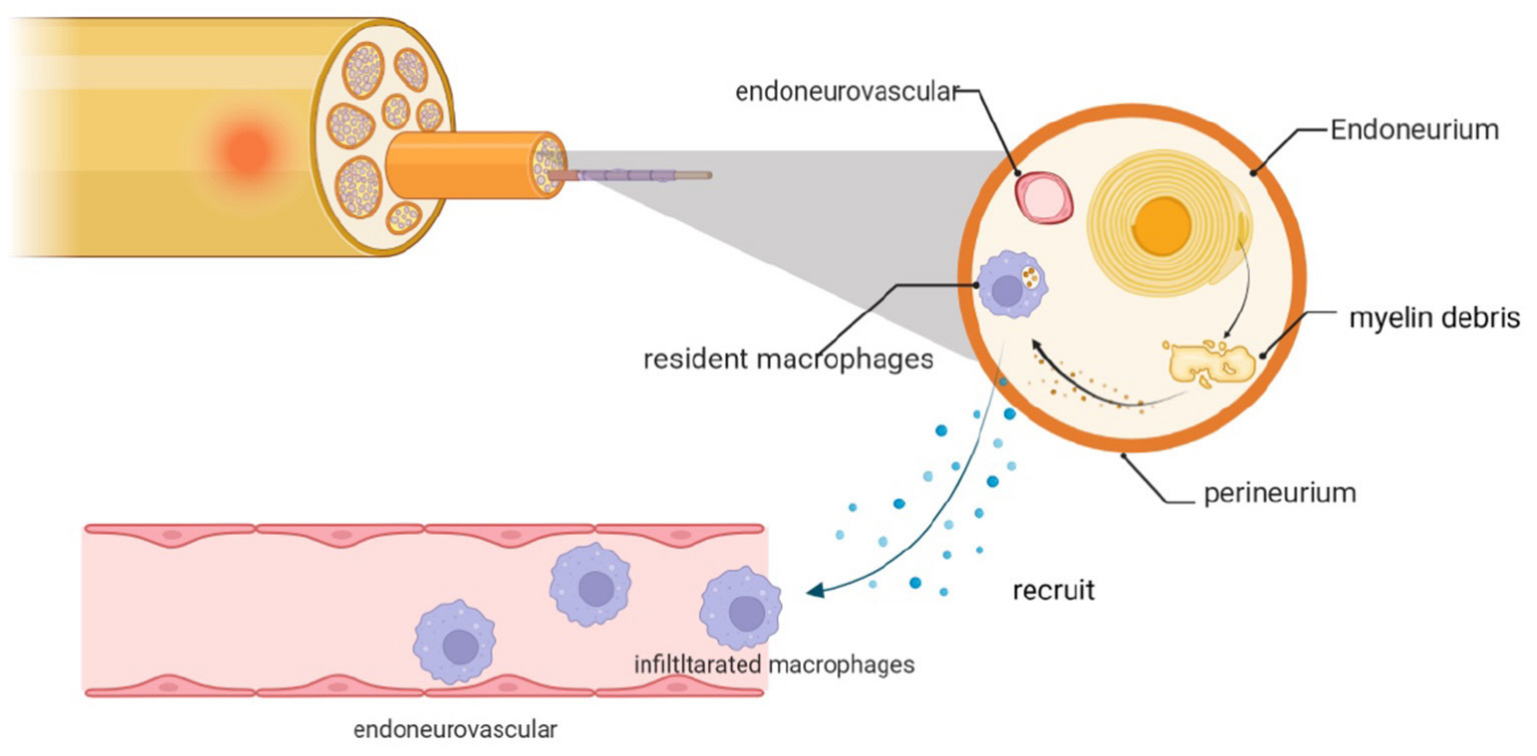
Myelin debris that accumulates after PNI is cleared by macrophages, including resident cells and those recruited locally from the neurovasculature. The infiltrating macrophages are recruited in response to chemokines and cytokines that are expressed by dedifferentiated SCs and resident macrophages.

After PNI, SCs will call on local macrophages and recruit cells from the peripheral circulation to assist with cleaning the microenvironment. Both resident and infiltrating macrophages provide critical contributions to this process (144) (Figure 4).

### Contribution of resident and infiltrating macrophages

#### Resident macrophages

Macrophages that are resident cells in peripheral nerves respond to injury and also play an active role in the early stages of peripheral nerve diseases (35, 145). Similar to SCs, resident macrophages are capable of phagocytosis, albeit to a more limited degree (23, 34). However, it is critical to recognize that all resident macrophages do not respond to all injuries. For example, a recent study by Ydens et al. revealed that primarily endoneurial macrophages respond to nerve crush injury (146). In addition to debris removal, resident macrophages secrete monocyte-attracting chemokines and can thus recruit additional macrophages and enhance debris clearance (36).

#### Infiltrating macrophages

More efficient macrophage recruitment and activation results in improved myelin clearance. Within the first 2 to 3 days after the disruption of the endoneurovascular, macrophages are recruited to and infiltrate the injury site. Macrophage accumulation reaches a peak at approximately 14 days post-injury (36). Circulating macrophages are attracted to the site of injury by inflammatory cytokines and chemokines, including TNF-α, interleukin (IL)-1α, IL-1β, chemokine C-C motif ligand 2 (CCL2), leukemia inhibitory factor (LIF), and pancreatitis-associated protein III (PAP-III) (36, 138, 147). Galectin-1 and galectin-3 are also critical factors involved in macrophage accumulation (147, 148); these mediators are expressed in SCs, nerves, and fibroblasts. Infiltrating macrophages also produce CCL2, TNFα, IL-1α, and IL-1β that collectively serve to augment recruitment and phagocytosis. Decomposition of the blood–nerve barrier (BNB) which isolates the nerve following the recruitment of macrophages is induced by Raf/ERK signaling in dedifferentiated SCs (69). A full discussion of several related mechanisms involved in the regulation of macrophage recruitment and activation is included in the sections to follow.

### Macrophages associated mechanisms promoting myelin clearance

#### Complement system

The complement system plays a critical role in modulating the impact of macrophages responding to a PNI. After an injury, the complement system can be activated through either classical or alternative pathways (149, 150). Complement component 3 (C3) and its receptor, complement receptor 3 (CR3), are central mediators of myelin clearance. CR3 expressed on the macrophage surface binds to the degenerating myelin sheaths and initiates phagocytosis (151). Phagocytosis is delayed in cells that have been treated with soluble (s)CR1, which is an inhibitor of both the classical and alternative pathways of complement activation (150). Recruitment and activation of macrophages are also suppressed in C3- (152), C5-, and C6-deficit mice (150, 153); myelin seems to remain intact in the C6-deficient strain (150). Collectively, these results suggest that the complement system contributes directly to SC- and macrophage-mediated myelin clearance.

#### Calcium-binding proteins S100A8 and S100A9

Calcium-binding proteins S100A8 and S100A9 activate inflammation and may initiate an acute phase response and a chemotactic gradient that precedes the main inflammatory response (154). Both S100A8 and S100A9 are upregulated in response to injury, specifically in both proximal and distal segments in nerve transection models. As these proteins are both toll-like receptor (TLR)4 ligands (155), their expression results in the induction of additional pro-inflammatory genes in SCs, including partial chemokines, inflammatory cytokines, and matrix metalloproteinases. These factors result in the infiltration of circulating macrophages at the injured site (154).

#### Toll-like receptors

Toll-like receptors (TLRs) are strongly induced in response to acute injury and play essential roles in the PNS (29). TLRs are expressed on the surface of resident macrophages and can be activated by endogenous ligands that initiate an innate immune response. Mice devoid of TLR2, TLR4, or myeloid differentiation factor 88 (MyD88) exhibited diminished macrophage recruitment due to impaired expression of CCL2 and IL-1β compared with their respective wild-type strains (156). The activation and recruitment of macrophages are augmented in response to the administration of TLR agonists, although this mechanism is impaired in mice that are TLR or TLR-signaling deficient. For example, Vallières et al. (157) reported that systemic injections of the TLR4 agonist, lipopolysaccharide (LPS), increased both macrophage recruitment and phagocytosis. TLRs respond to both pathogen-associated molecular patterns (PAMPs) (158) and to nonpathogenic ligands, including necrotic cells, heat shock proteins (HSPs 60 and 70), and extracellular matrix (ECM) components (159) that typically accumulate and promote inflammation at the site of a nerve injury site (160). TLR3, TLR4, and TLR7 also contribute to the immune surveillance of the PNS (29). Overall, TLRs provide critical signals that permit the PNS to respond to injury by activating macrophages and initiating myelin clearance.

#### G protein-coupled receptor Gpr126/Adgrg6

Gpr126/Adgrg6 has been characterized as a critical mediator of nerve development (161) and a regulator of myelin sheath formation (162). However, recent evidence suggests that this receptor plays an essential role in the recruitment of macrophages and ultimately myelin regeneration. Mogha et al. reported that Gpr126 contributed to the regulation of chemokines and TNF-α; levels of downstream targets, including CCL2, CCL3, and CXCL10, are reduced in Gpr126 deficit mice (163). These results suggest that the relationship between Gpr126 and chemokines has a significant impact on the regulation of macrophage recruitment. Notably, c-Jun, a factor known to be involved in regeneration, is upregulated in Gpr126-deficient mice. Therefore, we speculate that efforts to regulate Gpr126 may lead to new strategies for the treatment of PNI (27, 164) and that the Gpr126/Adgrg6 receptor may be targeted to promote the recruitment of macrophages and clearance of myelin debris.

#### Serum amyloid A

Serum amyloid A is a major component of the acute phase response to injury and infection that induces the production of pro-inflammatory chemokines. While SAA is synthesized primarily in the liver, the generation of SAA at extrahepatic sites may serve to regulate local inflammatory responses (165). SAA can be synthesized in SCs in response to injury and can induce the production of chemokines CCL2 and CCL12. SAA also has functions that are similar to those of chemokines and provides crucial contributions to macrophage recruitment. Expression of SAA is suppressed in IL-6-deficit mice in association with reduced levels of CCL2; these results suggest that SAA may be a key modulator of IL-6-mediated induction of this chemokine. Results from recent reports reveal that SAA is upregulated in SCs 3 days after injury (166) and that this response is significantly diminished in IL-6 gene-deleted mice. Collectively, these results suggest that IL-6 plays an essential role in SAA expression induced in response to injury (166) and that the IL-6-SAA-CCL2 pathway may be a new target that can be exploited therapeutically to regulate macrophage recruitment.

#### Apolipoprotein D

Apolipoprotein D is a glia-derived apolipoprotein that is required for a timely and effective response to injury (167, 168). As reported in a previous study, the role played by ApoD in response to nerve injury is associated with the dedifferentiation SCs (169). Myelin clearance and regeneration are delayed in injured nerves from ApoD-deficient mice; these results suggest that ApoD may play a crucial role in promoting myelin clearance. The report further demonstrated that the number of macrophages at the injury site was related to levels of ApoD, which in turn had a substantial impact on myelin clearance (169). The rates of both macrophage recruitment and myelin clearance were reduced in ApoD gene-deleted mice. Expression of the effector molecule, galectin-3, was also influenced by ApoD (169). The results of this study suggested a negative feedback mechanism in which ApoD regulated the production of factors associated with TLR activation, including CCL2 and TNF-α, and thus controlled for the potential over-recruitment of circulating macrophages (169). ApoD also helps to promote efficient myelin degradation; while myelin-positive ApoD-deficient macrophages accumulate more rapidly, these cells accumulate larger myelin particles and require more time to degrade MBP and MAG (169). Overall, the main impact of ApoD on myelin clearance relates to its capacity to recruit and activate macrophages and to promote autophagy *via* Becn1 (168). Thus, ApoD is another potential target that might be developed for new treatment strategies.

#### Sox2

The transcription factor, Sox2, limits the myelination of SCs both *in vitro* and *in vivo via* its capacity to suppress Krox20 (170). Sox2 also promotes clearance of myelin debris *via* its capacity to promote macrophage recruitment of macrophages. Long-term expression of Sox2 results in a persistent inflammatory state. Interestingly, while overexpression of Sox2 promotes both proliferation and radial sorting of SCs, it also limits SC-mediated myelination and thus inhibits functional recovery in the PNS (171). While these findings may seem contradictory, they can be understood in light of the development of PNS. Thus, Sox2 may also be a critical therapeutic target *via* strategies that precisely regulate its expression based on the time elapsed after the acute injury.

#### Fibroblast growth factor 9

Fibroblast growth factor 9 contributes to the recruitment of M1 macrophages and macrophage recruitment is diminished in FGF9-deficient mice. Ablation of FGF9 also limited the accumulation of CD68-positive and CD86-positive macrophages (172). Consequently, we speculate that FGF9 is a critical factor involved in clearing myelin debris after a PNI.

#### Phospholipase A2 family

The phospholipase A2 enzyme family includes a calcium-dependent group IVA (GIVA cPLA2) and a calcium-independent group VIA (GVIA iPLA 2). PLA2s regulate phospholipid metabolism, membrane turnover, host defense, and signal transduction (61, 173); these activities have been characterized in the PNS (174). PLA2s play important roles in regulating the metabolic activities of infiltrated macrophages. SCs express both group IVA and group VIA PLAs approximately 6 h after injury. López-Vales et al. (175) reported diminished expression of IL-1β and CCL2 mRNA in nerves devoid of group IVA and group VIA PLA2s in association with a reduction in the number of macrophages. Therefore, this mechanism also needs to be considered as a way to regulate macrophages.

#### Adhesion molecules

Intercellular cell adhesion molecule-1 (ICAM-1) is a member of the immunoglobulin superfamily that is upregulated in SCs and venous endothelial cells in the nerve tissue that promotes macrophage recruitment (176). ICAM-1 is upregulated in the early stages immediately after injury. Ablation of ICAM-1 results in decreased recruitment of macrophages in response to a transection injury as well as the accumulation of more myelin debris (177). However, this result was not confirmed in experiments performed using a different strain of ICAM-1 gene-deleted mice (178). Further study will be needed to confirm or refute the original findings.

P-selectin is another cell adhesion molecule that is expressed both on blood vessel endothelial cells and activated platelets. The interaction between P-selectin and its ligand, P-selectin ligand (PSGL-1), may promote macrophage recruitment and sustain this cell population at a later stage after injury (179). The expression of TNF-α, IL-6, and other pro-inflammatory cytokines was significantly attenuated in injured nerves from P-selectin-deficient mice (179). These results imply that P-selectin may be an essential mediator of macrophage infiltration.

#### Microvascular endothelial cells

Results from several recent studies revealed that microvascular endothelial cells are also crucial for the recruitment of macrophages and engulfing myelin debris that accumulates in response to injury (35, 180).

Macrophages are the cells that are primarily responsible for clearing myelin debris that accumulates in response to acute PNI. The processes involved in macrophage recruitment and activation are complex and in need of further research. Among the points not considered in this review, additional consideration of macrophage classification and the unique roles of M1 and M2 macrophages might be warranted. However, the M1 and M2 macrophage phenotypes cannot be determined accurately in cells and tissues from experiments performed *in vivo* (146). Here, we focused our attention on the activation and recruitment of macrophages under conditions that have been closely associated with myelin clearance. Interestingly, although macrophages are typically the primary mode of myelin clearance, disruption of macrophage recruitment has no clear impact on this process. Results from several studies revealed that neutrophils can also clear myelin debris and can compensate for the absence of macrophages in these circumstances (35). Fibroblasts may also have the capacity to remove tissue debris along with SCs during the early stages of the injury (4, 181).

## Exogeneous interventions

Numerous exogenous approaches, listed in Table 1, may be used to promote the clearance of myelin fragments. These approaches are based on the mechanisms associated with myelination (including negative regulation), autophagy, and the role of resident and infiltrating macrophages. These strategies will be discussed at length in the following article and may provide one or more new directions for research undertakings.

**Table 1:** Exogeneous interventions.

**Name**	**Intervention ways**	**Mechanism**	**References**
Nuclear factor κB (NF-κB)	Exogenous inhibition	Suppress myelination	(2, 3)
Ascorbic acid	Exogenous inhibition	Suppress myelination; Promote macrophage infiltration	(4)
Wnt/β-catenin	Exogenous inhibition	Suppress myelination	(5, 6)
Oxysterols	Treat with LXRs	Suppress myelination	(7)
Low-density lipoprotein receptor-related protein 4(Lrp4)	Exogenous inhibition	Suppress myelination	(8, 9)
LKB1 Liver kinase B1(LKB1)	Exogenous inhibition	Suppress myelination	(10)
HDAC1/2	Exogenous inhibition	Suppress myelination	(11)
SncRNA715	Not mentioned *in vivo*	Suppress myelination	(12)
circRNA.2837	Exogenous inhibition	Promote autophagy	(13)
Resveratrol (RSV)	Given exogenously	Promote autophagy	(14)
Rapamycin	Given exogenously	Promote autophagy	(15)
Epothilone B (EropB)	Given exogenously	Promote autophagy	(16)
Metformin	Given exogenously	Promote autophagy	(17)
β-Site amyloid precursor protein (APP) cleaving enzyme 1 (BACE1)	Exogenous inhibition	Promote the macrophages phagocytosis	(18, 19)
E6020	Given exogenously	Promote the macrophages phagocytosis	(20)
Signal regulatory protein-α (SIRPα)	Exogenous inhibition	Promote the macrophages phagocytosis	(21)

### Inhibition of NF-κB

Nuclear factor κB (NF-κB) is a master regulator of the inflammatory response and a mediator in many disease processes. While activation and translocation of NF-κB are essential to promote SC myelination and differentiation *in vitro* (182, 183), this factor is dispensable for myelination *in vivo* (3). Previous evidence suggested that NF-κB might be an essential mediator of PNI and that compact remyelination is delayed by short-term suppression of this transcription factor (2). Interestingly, NF-κB inhibition was found to promote regeneration after PNI (2). Therefore, factors that regulate NF-κB activation and translocation may be critical factors associated with the process of myelin removal.

### Ascorbic acid

Ascorbic acid, also known as vitamin C, is an essential micronutrient. Recent reports suggest that AA was also important for repairing nerve injury, specifically, both morphological and functional recovery of injured peripheral nerves (184). Other researchers reported that the administration of AA resulted in enhanced levels of c-Jun level and diminished levels of MAG during the early stages of PNI; collectively, these responses all repress myelin formation (4). AA also plays a critical role in macrophage recruitment. The results revealed that AA could enhance macrophage migration and infiltration and thus provide support for a suitable microenvironment (4). Interestingly, AA was also capable of promoting myelination *via* the activation of DNA demethylation (185). Collectively, these results suggest that AA may be an effective means to promote repair throughout the entire period after nerve injury.

### Inhibition of Wnt/β-catenin

The Wnt/β-catenin pathway regulates the expression of myelin-related genes (48). Wnt promotes the myelin gene expression by suppressing glycogen synthase kinase 3β (GSK3β); this will preserve β-catenin which then enters the nucleus. Once in the nucleus, β-catenin interacts with T-cell factor/lymphoid-enhancer factors (TCF/LEF or TCFs) that regulate the expression of myelin-related genes (79).

Notably, GSK3β is an essential factor in the pathway that regulates myelin-related gene expression. In addition, recent evidence suggests that early clearance of myelin debris is enhanced in response to lithium chloride (LiCl) which is a characterized inhibitor of the enzymatic activity of GSK3β (6). An evaluation of the subsequent phase revealed that myelin regeneration occurred in parallel with the findings described in the previous study (5). Thus, GSK3β may be another critical target for therapeutic strategies designed to promote the clearance of myelin debris and ultimately myelin regeneration. Accordingly, the GSK3β inhibitor LiCl may be applied in peripheral nerves as a bidirectional regulator of myelin synthesis. The roles of other GSK3β inhibitors remain to be explored.

### Oxysterols

Oxysterol is known for its impact on cholesterol homeostasis and in neurodegenerative disorders including Alzheimer's disease (186) and multiple sclerosis (187, 188). Oxysterol produced in SCs of PNS suppresses the expression of myelin-related genes, Pmp22, and MPZ in experiments performed *in vitro* (5). Oxysterol treatment of cells expressing high levels of liver X receptors (LXRs) also resulted in significant suppression; these results suggested that these inhibitory effects may be mediated by LXR ligands. Additional evidence revealed that LXR-mediated suppression might be the result of inhibition of the classical Wnt pathway (7). LXRs are likely to be involved in many pathways that promote both physiological and pathological responses in the PNS (189). The aforementioned mechanism might be clarified with additional research.

### Low-density lipoprotein receptor-related protein 4

Low-density lipoprotein receptor-related protein 4 is a critical protein that contributes directly to the Agrin-Lrp4-MuSK signaling pathway and is an essential regulator in nervous system development (8). Recent evidence revealed that Lrp4 expressed in SCs may be crucial for the regeneration of axons *via* an extrinsic mechanism (190). Moreover, Krox20 is downregulated in Lrp4 conditional gene-deleted mice together with low levels of MPZ (9). In this study, mutant mice exhibited superior repair properties and benefitted from the rapid clearance of myelin and proliferation of SCs that resulted from Krox20 downregulation after injury. Exploring a more defined mechanism of inhibition, Krox20 may support a new target for regenerating axons.

### Inhibition of liver kinase B1

Liver kinase B1 is asymmetrically localized at the SC–axon interface; this specific localization is dependent on PKA-mediated phosphorylation at Ser-431. LKB1 may be the central regulator of cellular asymmetry in SCs which is a property required to initiate myelination. Accordingly, the expression of Krox20 and MPZ is suppressed in LKB1 gene-deleted mice in association with delayed myelination (10). The LKB1-mutant mice demonstrated hypomyelination and limb handicaps (10, 191). In addition, the LKB1 deficit limits the activation of the tricarboxylic acid cycle (TCA) and thus reduces the production of citrate. Citrate is a six-carbon precursor to many cellular lipids; thus, changes in its intracellular concentration will have an immediate impact on normal myelination (191, 192). Therefore, we hypothesize that in the early stages after injury, LKB1 localization might be modulated by targeting PKA-mediated phosphorylation of Ser431, thereby regulating the process of myelin removal.

### Epigenetic modulators

Epigenetic modulators may have an impact on myelin-related gene expression and clearance. For example, the expression of histone deacetylase (HDAC)1/2 was strongly upregulated after PNI. The high expression levels of HDAC1/2 resulted in delayed demyelination and axon degeneration (11). Conversely, deletion of the genes encoding HDAC1/2 delayed the expression of Oct6 and enhanced c-Jun. These events helped to transform SCs into a pro-repair state and to reduce the expression of both Krox20 and MPZ. Moreover, recent results revealed that a brief period of HDAC1/2 inhibition promotes nerve regeneration together with the increased thickness of myelin. Thus, we conjecture that early inhibition of HDAC1/2 promotes regeneration by accelerating phenotype conversion and suppressing myelination during the early stages.

Recently, it has been found that histone deacetylase 3 (HDAC3, a histone-modifying enzyme) acts as an inhibitor of SC myelination (193). HDAC3 was proved with a high-level expression after injury in SCs to suppress the process of remyelination. This overexpression represses the activation of PI3K-AKT and ERK. HDAC3 cooperates with histone acetyltransferase (HAT) targeting TEAD4, described as a new inhibitor of SC myelin growth, which is shown by genomic occupancy analyses (193). Although partial inhibition of HDAC3 led to faster neurological and functional recovery, we currently do not know the contribution of HDAC3 to myelin clearance in the early phase after injury. Still, this possibility function cannot be denied. HDAC3 may play a bidirectional regulatory role in the process of neurological recovery, which needs to be demonstrated by further studies.

### Noncoding RNA

Small non-coding RNA 715 (sncRNA 715) originates from ribosomal DNA (rDNA) and was identified as an early inhibitor of demyelination and MBP translation in the CNS (194). Recent results documented that sncRNA 715 was expressed in pro-myelin SCs in PNS where it regulates MBP translation via a mechanism similar to that described in the CNS (12). Although we are not aware of any alterations in sncRNA715 expression in response to injury, we hypothesize that myelin removal might be regulated by exogenous interventions that target sncRNA715. Therefore, strategies that target noncoding RNA may be used to stabilize the microenvironment during the early stages of injury.

Circular RNAs (circRNAs) are non-coding RNAs found in the cytoplasm that regulate transcriptional and posttranscriptional gene expression. While there is little evidence available that documents the role of circRNA in the PNS, recent evidence suggests that they may be functionally associated with processes that regulate autophagy. Zhou et al. (13) reported significant discrepancies in the expression of circRNAs immediately after PNI; the elimination of circRNA.2837 resulted in the amplification of autophagy in primary spinal nerves. Likewise, the downregulation of circRNA *in vitro* could block static nerve injury by promoting autophagy *via* targeting miR-34a (13).

### Resveratrol

Resveratrol activates autophagy and thus has an impact on many signals that promote or alleviate pathology, including those contributing to nerve injury and CNS/PNS diseases. Although its mechanism of action remains unclear, RSV is believed to promote autophagy *via* its interactions with signaling pathways associated with mTOR, AMPK, SIRT1, PI3K/Akt, and MAPK. RSV also suppresses the TLR4/NF-κB signaling to activate autophagy, thereby accelerating clearance and promoting regeneration (14). Administration of RSV may thus be an effective strategy for the treatment of PNS and CNS injury.

### Rapamycin

In mammals, mTOR is a downstream factor of PI3K/Akt signaling (195) and regulates the formation of myelin and the growth of axons. Results from previous studies revealed that mTOR inhibition activates autophagy and thus promotes the clearance of damaged cellular components and preserves homeostasis (196–198). A study of PNI in rats reported that treatment with the mTOR inhibitor, rapamycin, promotes peripheral nerve regeneration and functional recovery after injury (15). While this may appear to be paradoxical given the role of mTOR in promoting negative regulation of myelin formation, autophagy detected in SCs under these circumstances may be the result of an mTOR-independent pathway. We suspect that this is likely given the timing of intervention after injury; in other words, timely intervention with mTOR inhibitors might enhance myelin clearance.

### Epothilone B

While EropB was initially used to treat cancer, additional studies revealed that this agent could promote recovery from CNS diseases (199, 200). The results of recent research suggest that EropB can aid with the structural and functional repair of damaged peripheral nerves (16). In contrast to the aforementioned strategy, EropB enhances autophagy in the PNS and promotes the migration of SCs. This process can be suppressed in the presence of the autophagy inhibitor, 3-MA. Additional research suggests that EropB may provide crucial regulation of this response by suppressing the PI3K/Akt pathway.

### Metformin

Metformin is a first-line anti-hyperglycemic agent used to treat type II diabetes mellitus. Metformin has also been used to relieve neurological problems, including neuropathic pain in the spine (201), cognitive decline, and memory loss (202). The results of a recent study reveal that metformin can induce autophagy in the early stages after injury and enhance the number of autophagosomes. This facilitates recovery by increasing the rate and extent of autophagy which results in more effective removal of myelin fragments (17).

### Inhibition of β-Site amyloid precursor protein cleaving enzyme 1

β-Site amyloid precursor protein cleaving enzyme 1 is an aspartyl protease known for its role in producing amyloid-β peptides in association with Alzheimer's disease. In the PNS, BACE1 cleaves neuregulin 1 type III (72) and, indirectly, APP (203). Reductions in BACE1 activity in gene-deleted rats enhance the clearance of myelin debris and promote axon regeneration compared to the wild-type (204, 205). By contrast, BACE1 over-expression leads to significant reductions in the length of the regenerated axons as well as the number of neuromuscular junctions (19). Treatment with a BACE1 inhibitor results in improved nerve regrowth and clearance of debris 7 days after a crushing injury (206). This rapid clearance is facilitated by the earlier influx of macrophages that are capable of more effective phagocytosis (79). The mechanisms underlying this response involve the upregulation of both tumor necrosis factor receptor 1 (TNFR1) and its downstream transcription factor, NF-κB, at the distal site of the injury.

#### E6020

E6020 is a lipid A mimetic and TLR4 agonist that promotes macrophage-mediated phagocytosis of accumulated debris. E6020 also induced cytokine production, activation of intracellular NF-κB signaling, and an overall “activated” macrophage morphology in experiments performed *in vitro*. Treatment with E6020 resulted in a larger macrophage infiltrate. Although these results cannot be paralleled with LPS, E6020-induced clearance might be controlled in the appropriate range and thus used to limit over-clearance (20).

#### Inhibition of signal regulatory proteinα

A SIRPα-dependent mechanism suppressed macrophage activation and thus impeded myelin clearance *in vivo*. Earlier studies revealed that CD47 associated with myelin interacted with SIRPα on macrophages to downregulate phagocytosis in the CNS (207). This interaction, combined with IL-10, constraints inflammation-induced macrophage phagocytosis and thus may protect healthy cells (208). Recent studies have characterized this interaction in the PNS. Injured rats undergoing SIRPα inhibition respond with rapid clearance of myelin, axon regeneration, and repair of the affected nerve. This effect results from blocking the binding between SIRPα and CD47 and removing CD47 from myelin fragments (21).

## Conclusion

Regeneration after PNI is influenced by many factors and molecular mechanisms. The extent of the injury and the ability to establish and provide ongoing support for a microenvironment that supports regeneration are among these critical features. Previous studies and clinical strategies have focused primarily on interventions that might promote axonal and myelin regeneration after injury; there has been little to no attention paid to the process of myelin clearance. Here, we discuss several strategies that might be used to promote myelin clearance. Collectively, current evidence suggests that clearance of myelin debris is beneficial for the process of regeneration during the early stages yet harmful if the process has been delayed. An improved understanding of mechanisms associated with myelin clearance after a PNI might contribute to new and effective therapeutic strategies to promote regeneration after injury. These findings will also provide essential contributions to our understanding of demyelinating neural disorders, degenerative diseases, and diseases of the CNS.

There are still many issues that need to be investigated, including the key pathways and the potential range of intervention strategies, as well as the aforementioned paradoxes and negative influences, among others. For example, one study features a nerve regeneration strategy involving partial enzymatic digestion of myelin debris. This mechanism facilitates the rapid removal of debris and creates a favorable microenvironment that has promoted the regeneration of damaged nerves in experimental rat models. However, as reported by Tsuang et al. (108), repair exhibited by the high-dose group was significantly less than that observed among those treated with lower doses. These results also suggest that treatment should be decided on a per-case basis and might focus on the rapid removal of cellular debris. Additional experiments will be needed to re-evaluate and optimize these strategies.

## Author contributions

Conceptualization: YY and YW. Investigation: YY and SW. Writing—original draft preparation: YY and MZ. Writing—review and editing, project administration, funding acquisition, and Supervision: YW. All authors have read and agreed to the published version of the manuscript.
